# Knockoff-Based Fine Mapping of MS-Associated SNPs in Sardinian Trios

**DOI:** 10.1007/s10528-025-11238-5

**Published:** 2025-08-30

**Authors:** Giulia Nicole Baldrighi, Andrea Nova, Claus Thorn Ekstrøm, Maria Luisa Piras, Maria Valeria Saddi, Luisa Bernardinelli, Teresa Fazia

**Affiliations:** 1 Department of Public Health, Experimental and Forensic Medicine, University of Pavia, 27100 Pavia, Italy; 2 Department of Brain and Behavioral Sciences, University of Pavia, 27100 Pavia, Italy; 3 Department of Public Health, University of Copenhagen, 1014 Copenhagen, Denmark; 4 Unità Operativa Neurologia, Stroke Unit, Ospedale S. Francesco – ASL 3 Nuoro, 08100 Nuoro, Italy

**Keywords:** Knockoff genotypes, Fine mapping, Family-based design, Complex disease, Multiple sclerosis

## Abstract

Genetic predisposition plays a key role in autoimmune and complex diseases such as multiple sclerosis (MS). However, identifying the specific variants or genomic regions responsible for disease susceptibility remains a significant challenge. In this study a family-based fine mapping approach was applied to analyze 142 trios, aiming to identify associated genetic variants linked to MS. The targeted genomic region resides within the 17:30,820,506–32,483,270 bp (Ch37/hg19), which includes the protein-coding gene ASIC2, previously implicated in MS and other neurological conditions, with surrounding genes comprising strongly correlated genetic variants to capture the broader signal from the region. Given the high prevalence of MS in Sardinia and the unique genetic characteristics of the Sardinian population, including reduced heterogeneity and extended linkage disequilibrium, we designed our study specifically within this population and focused on family-based data to enhance the power for detecting genetic signals, avoiding false discoveries. Genotype imputation found 2537 variants, which were then analyzed using the *knockoff Trio* method to identify loci associated with MS susceptibility. We found rs756787 (3′UTR of MYO1D) increased disease risk (OR 1.57, 95% CI [1.07–2.29], *p* = 0.02), while rs56175840 (intronic ASIC2) showed a protective effect (OR 0.17, 95% CI [0.04–0.74], *p* = 0.02), demonstrating the power of knockoff-based fine mapping in family datasets. Integrating LD-based expression and trait analyses helped reveal how rs756787 correlates with variants affecting genes involved in neurodegeneration and the immune response to Epstein–Barr virus, a known environmental factor implicated in MS pathogenesis. Our study highlights the effectiveness of knockoff-based fine mapping combined with expression-trait integration to identify genetic variants influencing MS risk in the Sardinian population.

**Supplementary Information:**

The online version contains supplementary material available at 10.1007/s10528-025-11238-5.

## Introduction

Multiple sclerosis (MS) is a chronic autoimmune disease attacking myelin, the layer covering neurons in the central nervous system (CNS) (Patsopoulos 2018). The dysregulation of the immune system allows cells to infiltrate and propagate inflammation, which gradually brings demyelination (Lemus et al. 2018) and irreversible disruption of electrical signal conduction. According to the complex nature, MS is defined as a complex disease caused by a combination of genetic, environmental factors (Jacobs et al. 2021), pathogenic agents, e.g., viruses and their interactions (Tarlinton et al. 2020). In this context, defining the genetic factors associated with MS is crucial for risk assessment and prediction models (Fazia et al. 2023; Brown et al. 2020). Investigating transmission in families with MS offers a powerful approach to identify both rare and common genetic variants contributing to disease risk, also studying the cumulative effect of common variants.

This paper focuses on a specific genomic region of chromosome 17 (30,820,506:32,483,270 bp, hg19) which includes acid-sensing ion channel 2 (ASIC2) protein-coding gene and nearby genes along with their regulatory elements. ASIC2 represent a widely expressed pH sensor in the brain thus acting in neuroinflammation process (Mango and Nisticò 2021). Its involvement in MS pathogenesis has been previously explored by performing in vivo and in vitro experiments (Fazia et al. 2019; McIlwrath et al. 2005), highlighting an increased expression of ASIC2 in human post-mortem brain MS samples compared to controls’ brain tissue. Based on this evidence, our research aimed at identifying genetic variants within this genetic region that may play a role in MS, clarifying its contribution to disease susceptibility and pathogenesis.

A family-based fine mapping analysis was performed on a sample of 142 trios from the genetically isolated region of Sardinia (Italy), a region characterized by an MS prevalence (330 per 100,000 inhabitants) higher compared to the mainland (122–232 per 100,000 inhabitants) (Battaglia and Bezzini 2017). Family studies offer the advantage of leveraging the power of genetic information given the common genetic background among the family members, enabling the detection of rare variants. Furthermore, families tend to be more homogeneous concerning exposure to environmental factors that can also be involved in the disease (Evangelou et al. 2006), reducing the confounding effects of environmental variability.

The adopted statistical framework is the KnockoffTrio (Yang et al. 2022), which is a knockoff-based framework for family trios. It offers key advantages over traditional genome-wide association study (GWAS) methods, particularly in family-based designs. Unlike the transmission disequilibrium test (TDT), which is robust to population stratification but limited in power and resolution in regions of high linkage disequilibrium (LD), KnockoffTrio enables fine mapping by separating true signals from LD-driven noise. It also improves performance of conventional methods that use conservative family-wise error rate (FWER) corrections by implementing flexible false discovery rate (FDR) control, boosting power while accounting for genetic correlation. Designed to address the limited power of trio-based studies with small sample sizes, KnockoffTrio enhances signal detection and reduces confounding, making it well-suited for family-based genetic analyses.

By systematically scanning multiple genomic windows at varying resolutions while accounting for LD structure, knockoff filtering strengthens signal detection of complex genetic landscapes (Vickerstaff et al. 2019). Finally, family-based logistic regression was performed to estimate the effect sizes of the most relevant independent signals, followed by comprehensive bioinformatic analyses, to pursue all the possible functional annotations, including proxy variants information, to highlight their biological relevance in the context of MS.

## Methods

### Sardinian Dataset

Participants were recruited from the Sardinian population using a combined trio-based and case–control design to maximize both analytical power and the resolution of the genetic architecture. Parent–offspring trios were prioritized from individuals with confirmed multi-generational Sardinian ancestry, predominantly sampled from historically isolated rural areas to capture the island’s distinctive and homogeneous genetic background. In parallel, unrelated cases and controls were enrolled for disease-focused association analyses, with careful representation across Sardinian subregions to account for known intra-island genetic stratification.

**Fig. 1:**
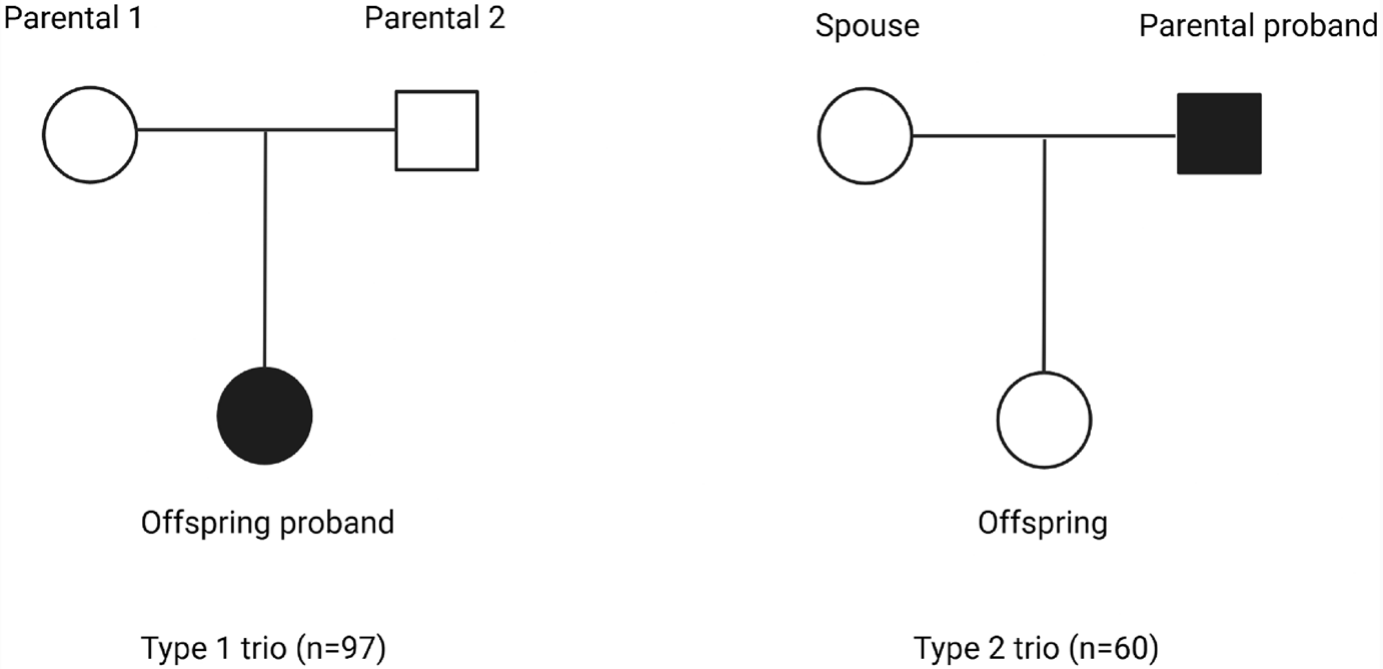
Diagram of the two types of trios included in the analysis. The proband in the figure (black), is referred to the MS patient in the family that serves as the starting point for the genetic study. On the left is shown the type 1 trio, where proband is the offspring, while on the right there is the type 2 trio where proband is one of the two parental individuals

The sample consisted of healthy controls and MS cases diagnosed according to Poser’s criteria (Seze and Bigaut 2021). Two different types of trios (Fig. 1) were included in the analysis: *type 1* (*n* = 97) composed by the proband and one or both parents, for a total of 291 subjects, and *type 2* (*n* = 60) composed of proband, spouse and a child, for a total of 180 subjects. The whole sample of 157 trios was used for the haplotype estimation and genotype imputation steps.

### Haplotype Estimation and Genotype Imputation

Genotypes were available for 99 genetic variants listed in Supplementary Table S1, covering the region from 32,493,488 to 34,156,251 bp. Haplotype estimation, i.e., phasing, was performed using Shapeit2 (Delaneau et al. 2008). The reconstructed haplotypes were used to impute missing variants in the region according to LD-based methods (Baldrighi et al. 2022) via Beagle5.4 software and following the pipeline by Browning (Browning and Browning 2011).

After haplotype estimation, input genotype data was first converted to VCF format with an extended window size to include a higher density of variants per genomic region, thereby improving the accuracy of haplotype inference. Imputation was conducted using a reference panel for haplotypes and a corresponding genetic map. To enhance population specificity, empirical allele frequencies derived from the study cohort were incorporated by modifying the reference panel’s file (legend), allowing Beagle to consider population-informed frequency estimates during imputation.

The accuracy of imputation was assessed through sensitivity analysis using *imputeqc* (Khvorykh and Khrunin 2020), by quantifying the discordance at each replication of the imputation, compared with a subset of complete and original data. At each replication, *imputeqc* uses the mask-reimpute algorithm, involving randomly spiking missing values within replicated datasets, while preserving the structure of the original ones, then re-imputing across replications to enhance accuracy. It ensures robustness by addressing missing data while preserving the integrity of replicated information. Post-imputation quality control (QC) was performed using PLINK (Purcell et al. 2007) considering a deviation from Hardy–Weinberg equilibrium with *p* < 10^−6^, minor allele frequency (MAF) < 0.01, thus including very rare variants only, genotype imputation rate at 0.01 and the presence of Mendelian errors.

### Genotype Matrix Augmentation via Knockoff Variables Generation and FBAT

**Fig. 2:**
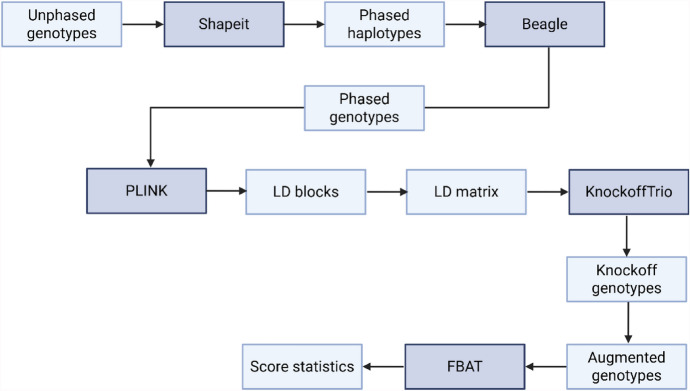
Workflow of the analysis. Tools used are highlighted in dark gray boxes: genotypes are phased by Shapeit and imputed by Beagle; linkage disequilibrium matrix and blocks are computed by PLINK, LD blocks are passed to KnockoffTrio to create knockoff genotypes, which are then combined with the real genotypes. Eventually, the family-based association test (FBAT), incorporated into KnockoffTrio, is used to compare the knockoff and real genotypes through a score statistic

The imputed matrix of genotypes was used for knockoff variable selection. Three steps were followed: (i) knockoff variable generation, augmenting the genotype matrix; (ii) weighted burden FBAT performed at multiple resolutions for identifying significant regions; (iii) family-based logistic regression for each relevant signal. The workflow is presented in Fig. 2.

*Knockoffs* (Bates et al. 2019) are variables designed to mimic the correlation structure found within the original variables, but independent of the disease under study. They serve as negative controls and allow the identification of significant signals, outperforming standard permutation methodology when correlation structure is predominant over association signals, as it happens in presence of LD. Knockoff framework has the advantage of mitigating the false positives’ issue and thereby improving the reliability of genetic findings. The matrix of genotypes (**X**) is augmented, because the knockoff variants’ matrix $\widehat{X}$ is added to the original one, hence the new matrix of genotypes (X*) is *augmented* (X* = [$X,$ $\widehat{X}$]). In a trio design, a pre haplotype reconstruction is required before creating knockoff genotypes, to adapt to family-wise hypothesis permutation. As explained by Yang et al. (2022), knockoff offspring haplotypes are generated given the phased haplotypes of the original trio for the considered region. By matching parental haplotypes with offspring haplotypes, the transmitted parental haplotypes to the offspring were firstly inferred. To estimate the knockoff haplotypes for the parents, the residual permutation method was used, based on the general sequential conditional independent pairs’ algorithm (Yang et al. 2022). Eventually, the knockoff offspring haplotypes are generated conditional on the knockoff parental haplotypes. Afterward, the weighted burden FBAT was applied on the augmented genotype matrix to perform the association scanning procedure at multiple resolutions, considering different window sizes (i.e., 500, 1000, 2000, 5000, 10,000, 15,000, and 20,000 bp) (Yang et al. 2022). Each of these windows overlaps with neighboring ones of the same size. The knockoff-based framework we employ here allows valid FDR control under arbitrary correlations.

The FBAT employed compares the observed transmission of alleles to offspring with the expected transmission under the generated knockoff. This test offers the advantage of accommodating scenarios where parents may also be affected by the disease as happens in our type 2 trio. Being a weighted burden FBAT instead of analyzing each variant individually, it aggregates information from multiple variants to evaluate their joint impact, assuming that they are associated with a trait with the same direction and magnitude of effect, returning a burden p-value (*p.burden*). Specifically, by implementing the LASSO regression a shrinkage hyperparameter is used, λ, defined as the L1 penalty, balances the trade-off between bias and variance in the determination of regression coefficients (Ranstam and Cook 2018). The Z score is computed considering variants’ burden test as in Eq. (1) where b is the regression coefficients of the linear combination which algebraically sum the effect of the variants in the burden test.

1 $${\mathrm{Z}} = {\text{argmin }}\left( {b\left( {0.5\left\| {y - Xb} \right\|^{2} 2 + \lambda \left\| b \right\|1} \right)} \right).$$

The same was calculated for the knockoff variants (Z*) across 10 repetitions. Eventually, the W score, which can be computed in several forms, was defined as the ratio W = Z/Z*, compared the signal of the original variants versus the knockoff ones. This approach improves detection power for sparse signals, with the number of knockoffs influencing the detection threshold. Since multiple comparisons are made between original genetic variants and knockoff variables, multiple testing correction methods are employed to control the overall false positive rate. Type I error α < 0.05 was considered as statistically significant. Lastly, a family-based logistic regression model was run for each available variant belonging to the prioritized window. The magnitude of its association with MS was estimated, in those trios in which at least one parent was in heterozygosity for the investigated variant. A post hoc power analysis was performed across different scenarios of MAFs and effect sizes using the R package *trio* (Neumann et al. 2014), assuming an additive model for our available numbers of trios.

### Proxy Variant Analysis Exploring LD-Based Trait Association, Tissue-Specific Expression, and Regulatory Elements

Relevant genetic variants identified within the knockoff framework were examined by performing a proxy analysis complemented by LD-based trait association and tissue-specific expression analyses, using LDlink and (Machiela and Chanock 2015), considering SNPs in nearby regions correlated with the studied variants. LDproxy provided information on proxy variants, including pairwise r^2^ values and functional annotations such as RegulomeDB and ForgeDB scores (Boyle et al. 2012). ForgeDB is a database that quantifies functional enrichment of variants in regulatory elements across diverse tissue and cell types, while RegulomeDB is a tool that scores SNPs based on their likelihood of affecting regulatory binding and function, with lower scores indicating stronger evidence for regulatory activity.

LD-based trait association used the GWAS Catalog, via dbSNP (GRCh37), to search for variants associated with traits or diseases. Variants were also investigated for associations with gene expression across different tissues using databases of expression quantitative trait locus (eQTL) to explore their regulatory effect on mRNA or protein levels. Furthermore, their relative biological pathways were investigated (Sherman et al. 2022).

### Cross-Population Exploration of Statistically Significant Variants

As a final step, to further validate the effect of the variants significantly associated to MS identified through our approach in the Sardinian trios and assess their relevance in broader cohorts of European individuals, we explored the summary statistics for the marginal SNPs-MS associations from the UK Biobank, obtained in 361,144 individuals of European ancestry (https://www.nealelab.is/uk-biobank), and from the International Multiple Sclerosis Genetics Consortium (IMSGC) (obtained in 115,803 individuals of European ancestry) (Elsworth et al. 2020).

## Results

### Genotype Imputation and Final Dataset

For the genotype imputation step, a sample of 471 individuals with 99 genotyped SNPs (Supplementary Table S1) in the investigated region was employed and 2537 variants were imputed in the candidate region 17:30,820,506–32483270 (full list is in Supplementary Table S2, available at https://github.com/giulnicole/SardinianTrios/tree/main/Supplementary_material).

Evaluation of discrepancy was performed through error rate, spanning from error rates 5 to 15% by 5%: the mean discrepancy ranged from 0.025% (with a standard deviation on the discrepancy sd = 0.0046%) to 0.061% (sd = 0.046%). On the imputed genotypes, QC was performed returning a final dataset of 2534 SNPs and 426 individuals belonging to 142 trios. In the Supplementary Table S3 the list of genes located in the imputed region is reported, while in Supplementary Fig. S1 the LD pattern is graphically described.

### FBAT Results on Augmented Knockoff Matrix

From the analysis it emerges that the strongest captured signals, compared to their knockoff, were in the windows (Table 1): (1) 30,821,006–30821505 bp, positively (i.e., risk role) associated with MS (Dir = 1), *p.burd*en = 0.0012. This window comprises one SNP, i.e., rs756787, located in the 3′UTR MYO1D and importantly, also comprises a portion of CDK5R1 gene. (2) 30,820,506–30821505 bp, positively associated with MS (Dir = 1), *p.burd*en = 0.004. This window comprises rs2249246, rs739455, and rs756787, all located in the 3′UTR MYO1D. (3) 31,421,506–31422005 bp, negatively (i.e., protective role) associated with MS (Dir = − 1), *p.burd*en = 0.0039. This window comprises the SNP rs56175840 an intron variant of ASIC2. (4) 32,322,506–32323005 positively (Dir = 1) associated with MS, *p. burd*en = 0.0047, comprising the SNP rs62056818, an intron variant of ASIC2. The effect size estimation for each of the relevant SNPs was obtained through a family-based logistic regression model, in those trios with heterozygous parents. Particularly, 140 trios were selected for the analysis of rs756787, 136 for rs56175840, 68 trios for the variant rs62056818. Results show that the T allele of rs756787 variant increases the risk of MS (OR 1.57, 95%CI [1.07,2.29], *p* = 0.02), while the T allele of the variant rs56175840 decreases the risk of MS (OR 0.17, 95%CI [0.04, 0.74], *p* = 0.02). Instead, rs62056818 (OR 2.00, *p* = 0.58), rs2249246 (OR 1.264, *p* = 0.20), and rs739455 (OR 2.00, *p* = 0.57) were not statistically significant associated with MS.

**Table 1:** Results of KnockoffTrio analysis

Chr	Start	End	n	Dir	W	p	Z	p.burden
17	30,821,006	30,821,505	1	1	2.005	0.002	3.242	0.001187887
17	31,421,506	31,422,005	1	− 1	2.040	0.0039	− 2.887	0.003892417
17	30,820,506	30,821,505	3	1	1.663	0.0040	2.637	0.004369347
17	32,322,506	32,323,005	1	1	1.468	0.0047	2.828	0.004677735

Table representing results from knockoff analysis. Four top-ranked genomic windows emerged from the analysis (p-value < 0.05). Chromosome, start and end of the significant region reporting signals are columns 1–3; column 4 indicates the number of SNPs impacting on MS presence according to the family-based association model; Direction (Dir) in column 5 indicates the direction of effect on MS presence, assuming value 1 for risk alleles and − 1 for protective effect of alleles. Column 6–8 contains the W ratio and *p*-value (*p*) calculated from the Z score as explicit in Eq. 1, and p.burden (column 9) indicates the p-value of the burden aggregation explained in *materials and methods* Sect. (1) 30,821,006–30821505 bp (*p* = 0.0012, risk) containing rs756787 (3′UTR of *MYO1D*) and part of *CDK5R1*; (3) 31,421,506–31422005 bp (*p* = 0.0039, protective) with rs56175840 (*ASIC2* intron variant); (3) 30,820,506–30821505 bp (*p* = 0.0040, risk) containing rs2249246, rs739455, and rs756787 (all in the 3′UTR of *MYO1D*); and (4) 32,322,506–32323005 bp (*p* = 0.0047, risk) comprising only rs62056818 (*ASIC2* intron variant)

A post hoc power analysis was conducted under an additive model, considering the sample of 142 trios and fixing the type I error rate at 0.05. Different scenarios were evaluated, by varying the MAF (0.05, 0.1, 0.2, 0.3) and effect sizes corresponding to ORs of 1.2 (small effect), 1.9 (medium effect), and 3.0 (large effect). Our results indicate that for small effect sizes (OR 1.2), the power remained low across all MAF values, ranging from 7.6% (MAF = 0.05) to 15.8% (MAF = 0.3). In contrast, for medium effect sizes (OR 1.9), power substantially increased, reaching 44.7% at MAF = 0.05 and 86.6% at MAF = 0.3. For large effect sizes (OR 3.0), our analysis showed high power, exceeding 92.9% at MAF = 0.05 and reaching nearly 100% at MAF = 0.2 and above.

### Proxy Variant Analysis Results for LD-Based Trait, Regulatory Elements and Tissue-Specific Expression

Results highlighted the protective role of rs56175840 and the risk role of rs756787. For these two genetic variants, proxy variants, LD-based traits association from previous GWAS and LD-based tissue-specific expression were investigated to explore their potential meaningful biological links with MS. In Table 2 are reported the lists of top-proxies in high LD (high LD threshold at r^2^ > 0.80) with rs756787 and rs56175840, respectively.

**Table 2:** Table of top-proxy variants of rs756787 and rs56175840

SNP	rs id (proxy)	Coord (chr17:bp)	Alleles	MAF	D′	r^2^	FORGEdb	RegulomeDB
**rs756787**	**rs8069868**	chr17:30,822,404	(T/C)	0.36	1	0.96	9	1d
	**rs8070040**	chr17:30,822,474	(T/C)	0.36	1	0.96	10	4
	**rs10853149**	chr17:30,823,382	(C/T)	0.36	1	0.96	9	1f
	**rs4795701**	chr17:30,824,081	(G/A)	0.35	0.98	0.93	8	5
	**rs2249246**	chr17:30,820,506	(T/G)	0.32	0.98	0.85	8	1f
**rs56175840**	**rs11080204**	chr17:31,420,382	(G/A)	0.18	1	0.97	4	4
	**rs4077500**	chr17:31,418,892	(G/T)	0.19	0.97	0.90	6	6
	**rs9916481**	chr17:31,417,207	(C/T)	0.19	0.97	0.90	6	5
	**rs9901562**	chr17:31,415,487	(C/A)	0.19	0.97	0.90	6	7
	**rs35858471**	chr17:31,413,372	(C/T)	0.19	0.97	0.90	4	5
	**rs34123551**	chr17:31,411,379	(C/A)	0.19	0.97	0.90	4	4
	**rs9895644**	chr17:31,409,231	(C/T)	0.19	0.97	0.90	6	5
	**rs9902094**	chr17:31,409,191	(T/A)	0.19	0.97	0.90	5	5
	**rs12603906**	chr17:31,408,515	(G/A)	0.19	0.97	0.90	5	5
	**rs11871218**	chr17:31,407,951	(G/A)	0.19	0.97	0.90	4	5

Top-proxy variants of rs756787 and rs56175840 (only showing SNPs in LD at r^2^ > 0.8). In the table are shown: rs id of each SNP, genomic coordinate on hg19, alleles, minor allele frequency (MAF), distance to the considered SNP for proxy analysis, D′ and r^2^ as correlation measures, functional relevance scores from FORGEdb and RegulomeDB

As shown in the table proxy variants reported pairwise r^2^ values ranging from 0.85 to 0.97 with the two variants identified relevant for MS (rs756787 and rs56175840). Functional annotations including RegulomeDB scores (1d-7) indicating regulatory potential and ForgeDB scores (4–10) suggesting mean-to-high functional relevance for most of the proxy variants of rs756787. RegulomeDB scores are ranked from 1 to 7, where scores of 1 d and 1f both indicate strong evidence for regulatory function, with 1 d featuring eQTL support plus transcription factor (TF) binding and DNase evidence, while 1f includes chromatin state evidence instead of eQTL data. While a score of 5 suggests minimal regulatory significance with limited support from functional studies, chromatin interactions, or eQTL data, typically for variants located in intronic regions, as it happens for most of the proxy SNPs of rs56175840.

Proxy variants were used to explore key relationships in this genomic area to find potential functional significance. For the LD-based trait analysis, considering all traits with a r^2^ > 0.20, the most interesting results were obtained for rs756787 given the associations with systolic blood pressure, smoking initiation, schizophrenia, reactive lymphocyte count, reactive lymphocyte percentage of white cells (Liu et al. 2019; Daskalakis et al. 2021) (Supplementary Table S4). For rs56175840 the only associated trait was BMI, but with a low LD coefficient (r^2^ = 0.1).

Moreover, the two SNPs were investigated for their involvement in the regulation of transcription factors, but no relevant results were found. The RegulomeDB analysis for chr17:32,494,365–32,494,366 reports 236 peaks, indicating overlapping regulatory features such as transcription factor binding sites, chromatin marks, or open chromatin regions, and a 1f rank, indicating a likely regulatory function with potential effects on eQTL and TF binding.

The genetic variant rs756787 showed a strong association with rs2249246 (r^2^ = 0.85), which is a variant associated with regulatory elements (IKZF1, ZNF263, CEBPA, IKZF2) interconnected with TAL1 in hematopoietic differentiation (GO:0030097), spinal cord dorsal interneuron differentiation (GO:0021527) and immune system development. The 3′UTR of MYO1D region in which rs756787 is located overlaps with the regulatory segment of two other protein-coding genes, i.e., PSMD11 and CDK5R1, respectively, known for involvement in pathways related to Epstein-Barr virus (EBV) lymphocytes modification and multiple neurodegenerative disease. By performing an *LDlink* analysis using *DAVID,* the rs756787, occurs to be in high LD (r^2^ > 0.95) with rs8069868 and rs8070040, two variants within the regulatory regions of PSMD11 and CDK5R1 (Table S5).

The lncRNA is associated with multiple tissues, including fibroblasts, lung, brain, and skin, Subsequently, from the LD-based tissue-specific expression analysis, considering variants in LD, no results were retrieved for rs56175840; while rs756787 resulted to be associated with the expression of CDK5R1, PSMD11, RHBDL3, lncRNA (ENSG00000274341.1), MYO1D and C17orf75 in different tissues (Supplementary Table while RHBDL3 shows significant negative correlations in brain regions; PSMD11 and C17orf75 exhibit tissue-specific expression in testis and nerves, and MYO1D and CDK5R1 are linked to arterial and blood tissues with both positive and negative correlations.

### Cross-Population Replication Using UK Biobank and IMSGC Summary Statistics

**Fig. 3:**
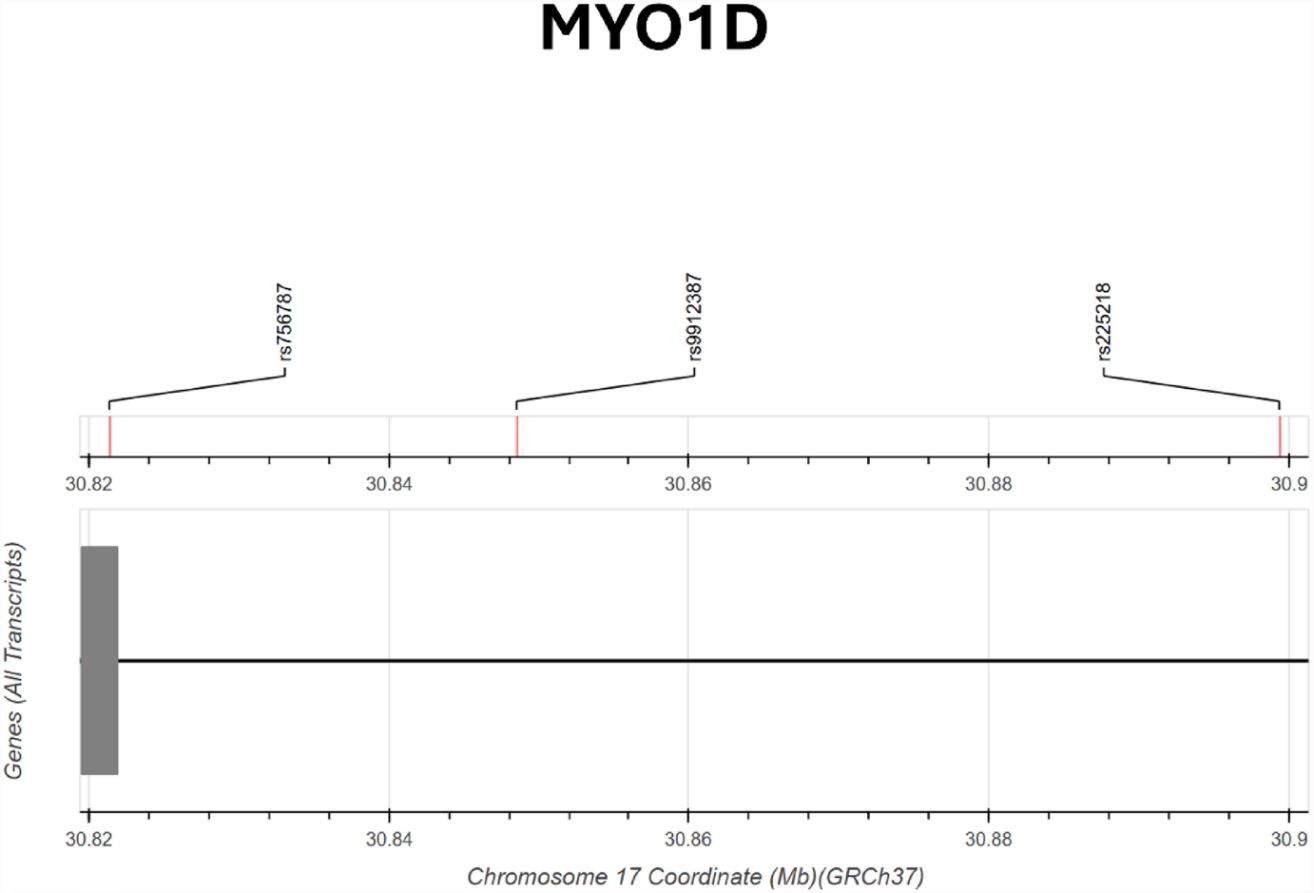
Genomic position of SNPs (from UK Biobank and IMSGC) associated with MS and their distance with rs756787 on chromosome 17 (30.82 Mb to 30.90 Mb, GRCh37). The SNPs rs9912387 (MYO1D intron) and rs225218 (MYO1D intron) are shown in low LD with rs756787 (r^2^ = 0.09 and 0.01; D′ = 0.36 and 0.17, respectively). All SNPs are plotted according to their genomic coordinates and annotated relative to all gene transcripts in the region

**Fig. 4:**
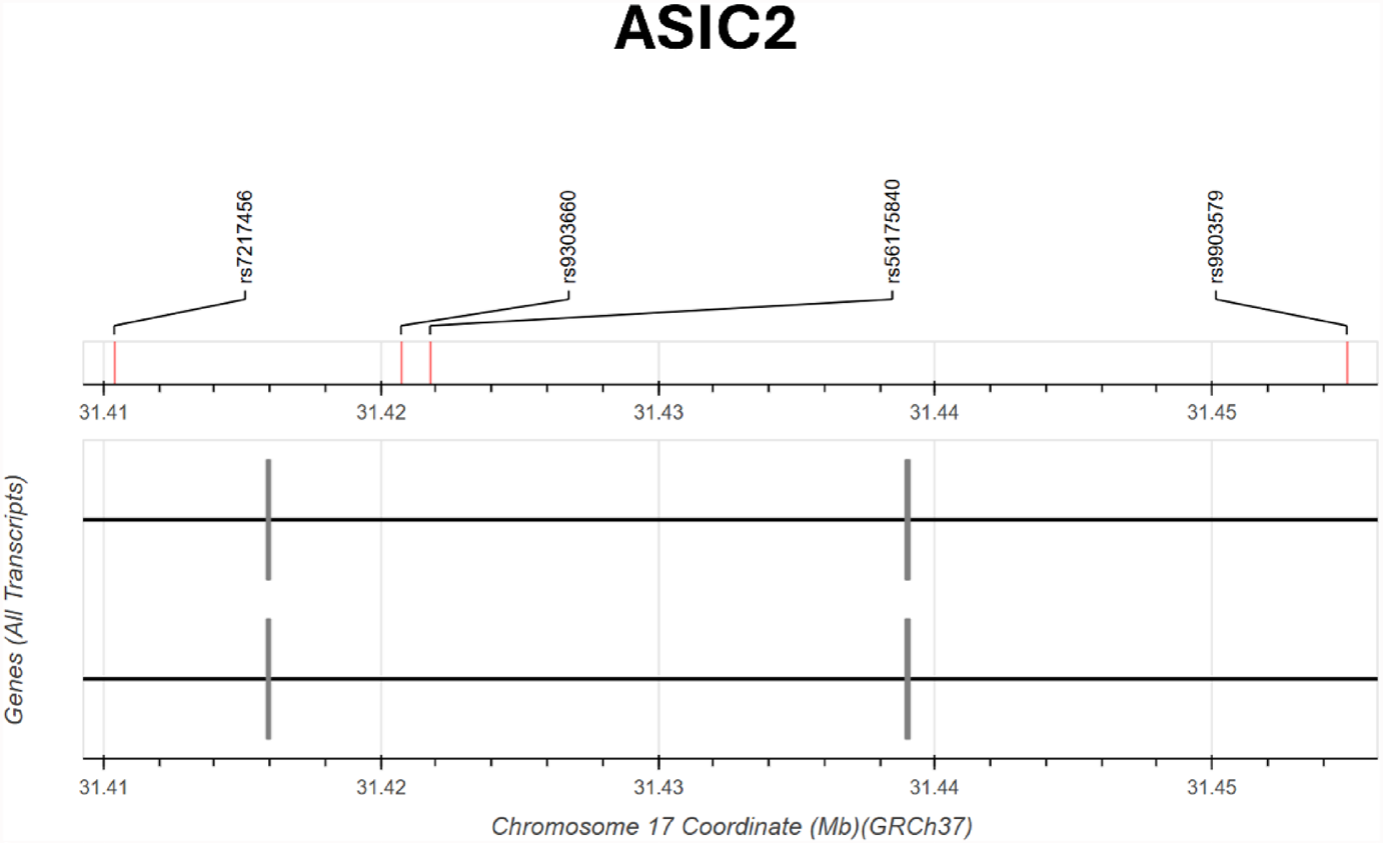
Genomic location of SNPs (from UK Biobank and IMSGC) associated with MS and their distance with rs56175840 on chromosome 17 (31.41 Mb to 31.46 Mb GRCh37). The intronic variants rs9303660 and rs7217456 in ASIC2 show moderate LD with rs56175840, while rs9903579 (ASIC2 intron) is in low LD with rs56175840 (r^2^ = 0.03; D′ = 1). SNPs are displayed by chromosomal position and gene context based on all transcript annotations

The effects of SNPs rs756787 and rs56175840 were explored in the summary statistics from UK Biobank and IMSGC, which contained marginal associations with MS in individuals of European ancestry. While the effect of both variants was not statistically significant in this cross-population comparison, closely located SNPs significantly associated with MS (*p* < 0.05) were found in low to moderate LD (r^2^ < 0.25 and D′ < 0.85) with rs756787 and rs56175840 (Figs. 3 and 4). Specifically, in the UK Biobank two SNPs (rs9303660 and rs7217456), both intron variants of ASIC2, in moderate LD with rs56175840 and a single SNP (rs9912387), an intron variant of MYO1D, in low LD with rs756787 (r^2^ = 0.09 and D′ = 0.36). From the IMSGC summary statistics were found the SNP rs225218, set in the intron of MYO1D, in low LD with rs756787 (r^2^ = 0.01 and D′ = 0.17), and the SNP rs9903579, set in the intron of ASIC2, in low LD with rs56175840 (r^2^ = 0.03 and D′ = 1 with rs56175840). Interestingly, both rs225218 and rs9912387 (in LD with rs756787) were also eQTLs for CDK5R1 expression in the blood, with rs225218 and rs9303660 being also eQTLs for MYO1D expression in the blood.

## Discussion

Understanding the genetic architecture is essential for improving risk assessment and prediction models for complex diseases. In studies involving related individuals, genetic similarities within families can amplify effect sizes compared to analyses with unrelated samples. However, family-based designs often suffer from limited sample sizes, reducing statistical power. KnockoffTrio strengthens the detection of genetic associations, improving robustness even in small family-based cohorts.

In this trio study, two variants were prioritized for their associations with MS: the variant rs756787, located in the 3′UTR of MYO1D, with previous evidence of LD-trait association (r^2^ > 0.20) to traits linked to MS (systolic blood pressure, smoking, reactive lymphocyte) (Liu et al. 2019; Daskalakis et al. 2021), suggesting a potential pleiotropic effect of this variant, while, rs56175840 in the intronic region of ASIC2, which did not report strong evidence of LD-trait association, only with BMI (r^2^ = 0.1).

The RegulomeDB analysis for chr17:32,494,365–32,494,366 provides more evidence regarding the involvement of rs756787 in regulation of expression: the score for peaks (236 peaks) indicates overlapping regulatory features, such as transcription factor binding sites, chromatin marks, or open chromatin regions. The 1f rank indicates a likely regulatory function, i.e., eQTL + TF binding/DNase peak possible to affect binding. For rs56175840, no results were found for regulatory elements.

Moreover, eQTL analysis was performed highlighting that only rs756787 showed strong correlations (r^2^ > 0.8) with variants within PSMD11 (rs8069868) and CDK5R1 (rs8070040) genes. These two variants were found to regulate PSMD11, implicated in EBV pathway regulation (Maier et al. 2006), which is a well-known for MS risk, CDK5R1, a neurodegenerative disease regulator (Ao et al. 2022) and MYO1D expression and transcription, already described for the implication in the crossing of the blood–brain barrier (BBB) (Duan et al. 2023). Indeed, Duan et al. (2023) and Maier et al. (2006) reported that MYO1D may play a crucial role in mediating the transfer of α-synuclein preformed fibrils (α-syn PFF) from the bloodstream into the brain. These aggregates can cross the BBB and enter the CNS, and MYO1D appears to facilitate their intercellular propagation. Therefore, the inhibition of MYO1D expression might block the transfer of α-syn PFF from brain microvascular endothelial cells to pericytes, suggesting a role for MYO1D in maintaining BBB integrity.

CDK5R1 gene encodes *p35*, the main activator subunit of cyclin-dependent kinase5 and the *p35/CDK5* complex plays a fundamental role in brain development and functioning (Moncini et al. 2017). Connections between *p35* and *Sod1,* encoded by the superoxide dismutase-1 (SOD1) were found through the mis-regulation of endoplasmic reticulum and the formation of free radicals/reactive oxygen species; *Sod1* has been found to be elevated in lesions of adaptive response to oxidative stress (Hensley et al. 2002), with the *Fadd protein,* encoded by the Fas-associated protein with death domain (FADD) turned out to have a crucial role in the regulation of apoptosis. It recruits the caspase-8/caspase-10 to the activated *Fas* or *Tnfr-1* receptors. *Sod1* and *Fadd* protein levels, measured in plasma of Sardinian families with MS history, were estimated to have a high additive genetic component with respective estimates ℎ^2^ = 0.58; 95%CI [0.18, 0.96] and ℎ^2^ = 0.41; 95%CI [0.06, 0.84] (Nova et al. 2022). PSMD11 encodes for *Psmd11* (Proteasome 26S subunit, non-ATPase 11), a subunit of the 19S regulatory particle of the 26S proteasome complex, responsible for recognizing and binding ubiquitinated proteins targeted for degradation. Some mis-recognition of the protein domains could compromise the proteasome’s function, affecting the ability of *Psmd11* and other proteasome subunits to recognize and degrade *Ebna1* (Epstein-Barr Nuclear Antigen1), thereby contributing to its stability and persistence in EBV-infected cells (Pinto et al. 2020). Infectious mononucleosis, caused by primary EBV infection, increases the risk of developing MS (Soldan and Lieberman 2023), and it has been shown that MS patients have antibodies that bind to both a protein from EBV and a protein made in the brain and spinal cord, suggesting that part of the virus’s protein mimics a protein in the CNS, leading to autoimmune system attack.

When examining marginal associations between rs756787 and rs56175840 and MS in UK Biobank and IMSGC summary statistics, neither variant showed statistically significant effects. However, nearby SNPs located within the introns of MYO1D and ASIC2, though in LD with our variants, were significantly associated with MS, supporting the potential involvement of these genes in disease risk. It is important to emphasize that marginal association results are not directly comparable to fine mapping outputs, which reflect conditional effects. Additionally, population-specific genetic differences between Sardinian and broader European populations, such as LD patterns, may explain discrepancies in association signals. These variants might represent risk factors specific to or enriched within the Sardinian population due to genetic isolation, founder effects, or unique environmental exposures.

Although causal relationships remain uncertain, our results derive from a fine mapping approach that offers improved resolution over standard GWASs within targeted genomic windows. Despite the interesting results, the study has limitations: (i) limited power to detect variants with small effect sizes due to sample size constraints; (ii) absence of phenotypic data beyond MS status, limiting the opportunity to investigate gene–environment interaction analyses; and (iii) reliance on bioinformatic tools to investigate correlations between rs756787 and rs56175840 and traits linked to MS, which require confirmation using advanced statistical methods, such as colocalization and Mendelian Randomization, and functional studies. Future studies with larger samples, broader phenotypic data, and functional validation, will be fundamental to clarify their potential mechanistic relevance to MS.

## Conclusion

In this family-based trio study, rs756787 (3′UTR of MYO1D) was prioritized for its strong regulatory associations with MS-relevant traits and genes (CDK5R1, PSMD11), suggesting involvement in neuroinflammation, BBB integrity, and EBV response. In contrast, rs56175840 (intronic region of ASIC2) showed no strong regulatory evidence, highlighting rs756787 as the more biologically plausible variant contributing to MS risk in this isolated population.

While causality remains unproven, our integrative genomic and bioinformatic approach supports growing evidence implicating EBV in MS pathogenesis and reinforces the rationale for exploring antiviral and immune-modulatory strategies as potential therapeutic avenues.

## Supplementary Information

Below is the link to the electronic supplementary material.

Supplementary file1 (PNG 463 KB)

Supplementary file2 (PNG 322 KB)

Supplementary file3 (DOCX 32 KB)

## Data Availability

The datasets analysed for this study can be found in the Github repository https://github.com/giulnicole/SardinianTrios.
