# Supplementary figures and images for: Knockoff-Based Fine Mapping of MS-Associated SNPs in Sardinian Trios
Source: Biochem Genet. 2025 Aug 30;64(3):4130–46. doi: 10.1007/s10528-025-11238-5 (PMC13186870; doi:10.1007/s10528-025-11238-5)

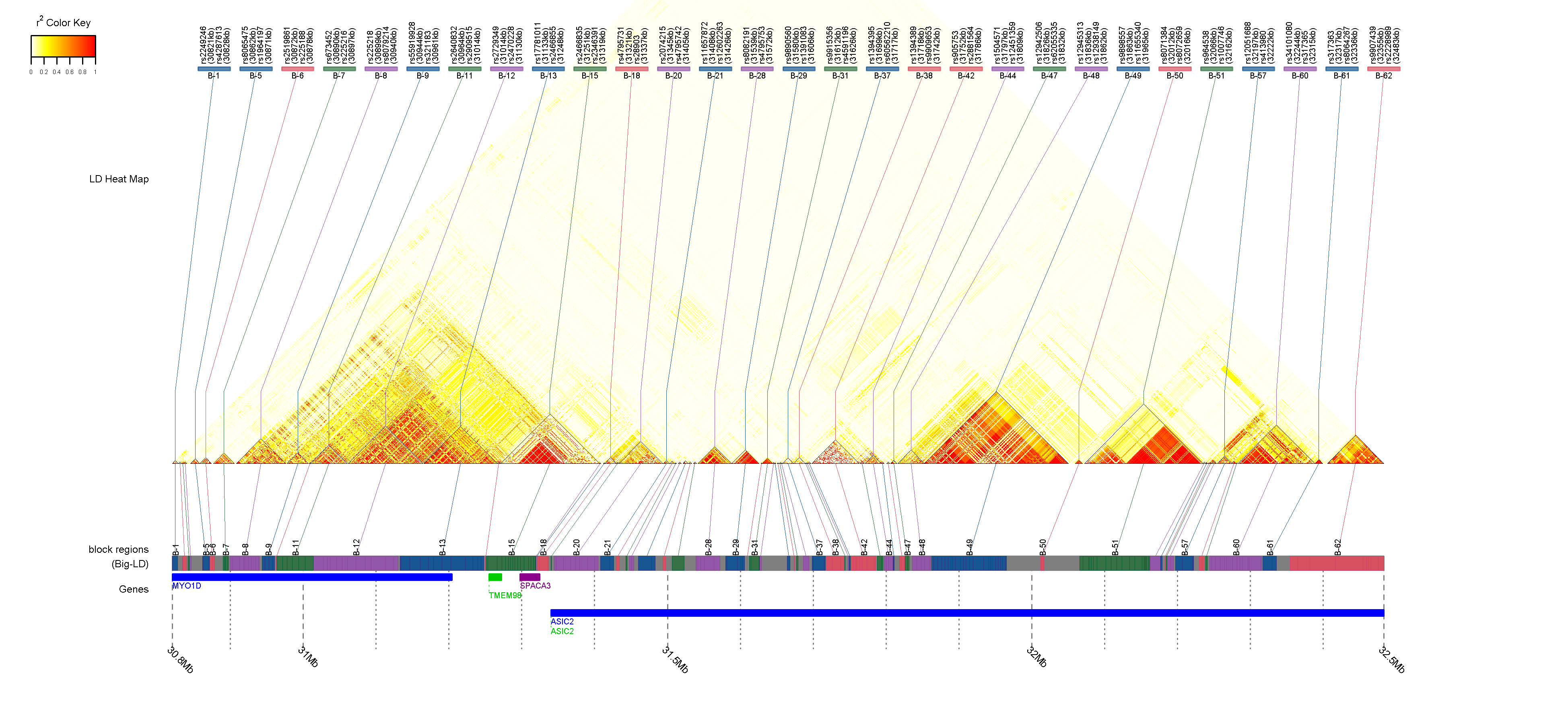

Supplement: Supplementary file 1 — Supplementary file1 (PNG 463 KB) [file 10528_2025_11238_MOESM1_ESM.png]

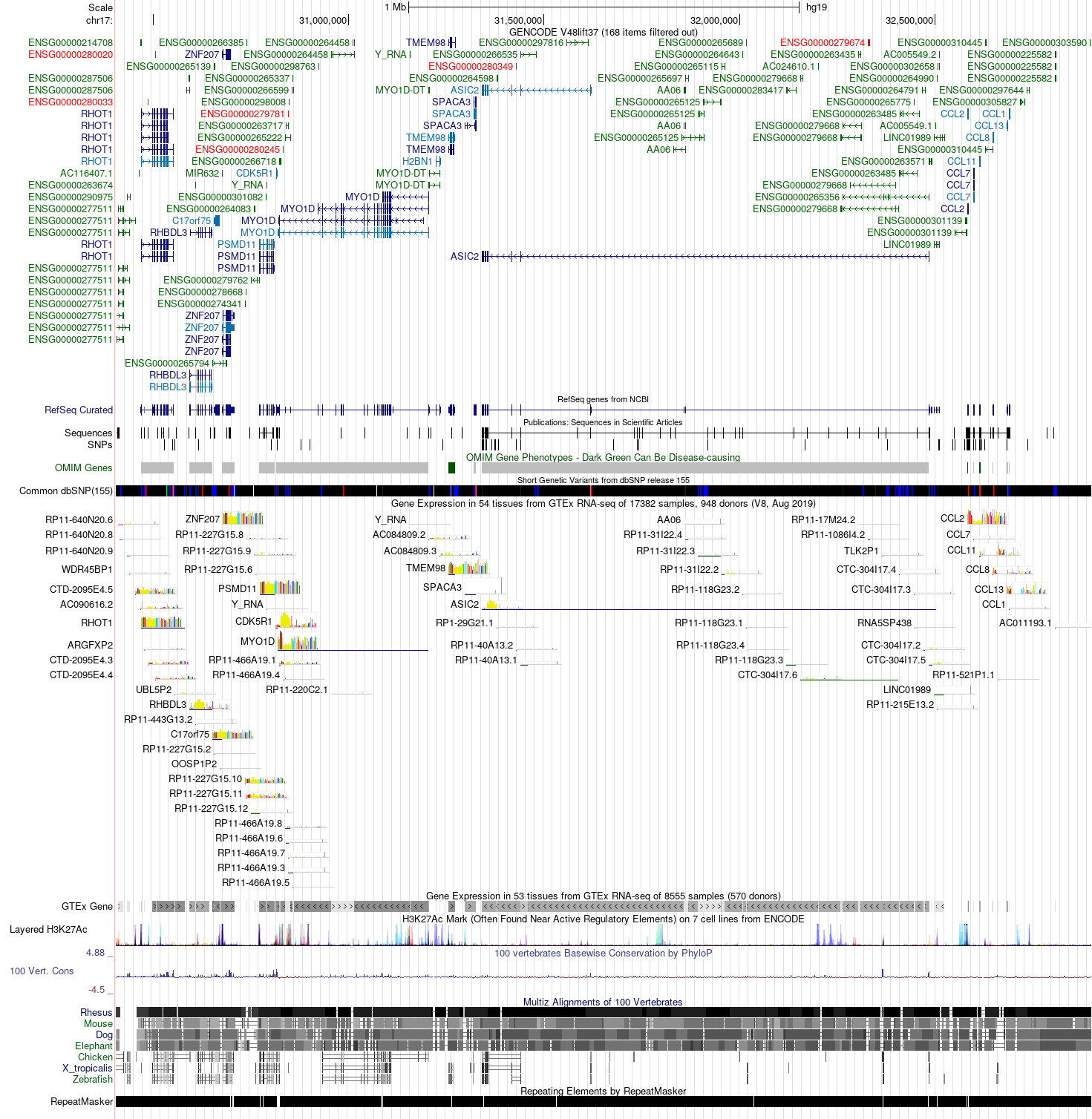

Supplement: Supplementary file 2 — Supplementary file2 (PNG 322 KB) [file 10528_2025_11238_MOESM2_ESM.png]
