# Supplementary material for: Knockoff-Based Fine Mapping of MS-Associated SNPs in Sardinian Trios
Source: Biochem Genet. 2025 Aug 30;64(3):4130–46. doi: 10.1007/s10528-025-11238-5 (PMC13186870; doi:10.1007/s10528-025-11238-5)
Supplement: Supplementary file 3 — Supplementary file3 (DOCX 32 KB) [file 10528_2025_11238_MOESM3_ESM.docx]

**Table. S1.** Table of SNPs from the Sardinian sample

| **Chr** | **SNP id** | **Gene** | **BP** |
| --- | --- | --- | --- |
| 17 | rs2249246 | CDK5R1 | 30820506 |
| 17 | rs2905171 | MYO1D | 30843673 |
| 17 | rs7212466 | MYO1D | 30844384 |
| 17 | rs9890602 | MYO1D | 30880382 |
| 17 | rs225186 | MYO1D | 30880899 |
| 17 | rs225212 | MYO1D | 30896455 |
| 17 | rs225215 | MYO1D | 30896910 |
| 17 | rs11654699 | MYO1D | 30903310 |
| 17 | rs7225208 | MYO1D | 30922784 |
| 17 | rs11868639 | MYO1D | 30934655 |
| 17 | rs321183 | MYO1D | 30960537 |
| 17 | rs1979706 | MYO1D | 30974499 |
| 17 | rs2249638 | MYO1D | 30980972 |
| 17 | rs2543970 | MYO1D | 30983784 |
| 17 | rs11650840 | MYO1D | 31040965 |
| 17 | rs4293434 | MYO1D | 31072993 |
| 17 | rs17183295 | MYO1D | 31078272 |
| 17 | rs10853153 | MYO1D | 31082863 |
| 17 | rs10512439 | MYO1D | 31107845 |
| 17 | rs9890129 |  | 31232773 |
| 17 | rs2470207 |  | 31237993 |
| 17 | rs2470209 |  | 31239329 |
| 17 | rs10512441 |  | 31239645 |
| 17 | rs12952271 | TMEM98 | 31255616 |
| 17 | rs28923 | TMEM98 | 31261649 |
| 17 | rs29004 | TMEM98 | 31264480 |
| 17 | rs28919 | TMEM98 | 31268111 |
| 17 | rs9772 | TMEM98 | 31268378 |
| 17 | rs11851 | TMEM98 | 31268429 |
| 17 | rs28918 | TMEM98 | 31269320 |
| 17 | rs28985 |  | 31279334 |
| 17 | rs7225406 |  | 31281356 |
| 17 | rs28913 |  | 31289779 |
| 17 | rs1557752 | SPACA3 | 31297486 |
| 17 | rs28909 | SPACA3 | 31303368 |
| 17 | rs7359655 | SPACA3 | 31306153 |
| 17 | rs4999077 | SPACA3 | 31318829 |
| 17 | rs2188952 | SPACA3 | 31319092 |
| 17 | rs28906 | SPACA3 | 31319764 |
| 17 | rs7225146 | SPACA3 | 31320785 |
| 17 | rs4795731 | SPACA3 | 31321391 |
| 17 | rs28964 | SPACA3 | 31322254 |
| 17 | rs28958 | SPACA3 | 31324657 |
| 17 | rs28957 | SPACA3 | 31324673 |
| 17 | rs28955 | SPACA3 | 31324976 |
| 17 | rs757079 | SPACA3 | 31325103 |
| 17 | rs28903 | ASIC2 | 31336980 |
| 17 | rs28936 | ASIC2 | 31340390 |
| 17 | rs28935 | ASIC2 | 31340444 |
| 17 | rs28933 | ASIC2 | 31340682 |
| 17 | rs2074215 | ASIC2 | 31344549 |
| 17 | rs16561 | ASIC2 | 31365752 |
| 17 | rs16586 | ASIC2 | 31376188 |
| 17 | rs16582 | ASIC2 | 31377248 |
| 17 | rs223031 | ASIC2 | 31399596 |
| 17 | rs4077500 | ASIC2 | 31418892 |
| 17 | rs11080204 | ASIC2 | 31420382 |
| 17 | rs3928996 | ASIC2 | 31422176 |
| 17 | rs7215616 | ASIC2 | 31449992 |
| 17 | rs319768 | ASIC2 | 31523167 |
| 17 | rs319759 | ASIC2 | 31561365 |
| 17 | rs319751 | ASIC2 | 31569677 |
| 17 | rs1553040 | ASIC2 | 31571194 |
| 17 | rs1002317 | ASIC2 | 31571560 |
| 17 | rs9915774 | ASIC2 | 31600299 |
| 17 | rs10459963 | ASIC2 | 31623326 |
| 17 | rs377066 | ASIC2 | 31644795 |
| 17 | rs1354492 | ASIC2 | 31673938 |
| 17 | rs2347155 | ASIC2 | 31737421 |
| 17 | rs7222667 | ASIC2 | 31773568 |
| 17 | rs4305127 | ASIC2 | 31800527 |
| 17 | rs12941035 | ASIC2 | 31817874 |
| 17 | rs4795800 | ASIC2 | 31847528 |
| 17 | rs964459 | ASIC2 | 31847693 |
| 17 | rs9899597 | ASIC2 | 31888205 |
| 17 | rs4794962 | ASIC2 | 31895597 |
| 17 | rs11080230 | ASIC2 | 31919979 |
| 17 | rs17782997 | ASIC2 | 31926374 |
| 17 | rs1984634 | ASIC2 | 31961260 |
| 17 | rs12951386 | ASIC2 | 31967460 |
| 17 | rs3115684 | ASIC2 | 31994232 |
| 17 | rs8078646 | ASIC2 | 32001745 |
| 17 | rs1490922 | ASIC2 | 32013255 |
| 17 | rs8067435 | ASIC2 | 32052655 |
| 17 | rs2637365 | ASIC2 | 32052862 |
| 17 | rs9896421 | ASIC2 | 32077378 |
| 17 | rs2881782 | ASIC2 | 32108304 |
| 17 | rs2881844 | ASIC2 | 32159519 |
| 17 | rs12936122 | ASIC2 | 32256079 |
| 17 | rs9904289 | ASIC2 | 32283478 |
| 17 | rs4493111 | ASIC2 | 32293030 |
| 17 | rs3744516 | ASIC2 | 32321054 |
| 17 | rs1808193 | ASIC2 | 32366000 |
| 17 | rs12449864 | ASIC2 | 32366547 |
| 17 | rs7207803 | ASIC2 | 32378513 |
| 17 | rs17193396 | ASIC2 | 32454246 |
| 17 | rs2228990 | ASIC2 | 32468982 |
| 17 | rs2228989 | ASIC2 | 32483237 |

Original genotyped SNPs (SNP id) in the Sardinian population, covering the region from 30820506 bp to 32483237 bp; gene name and bp are reported according to Ch37 (hg19).

**Table. S2.** BIM file of the imputed variants (2537 SNPs)

Available at: https://github.com/giulnicole/SardinianTrios/tree/main/Supplementary_material

**Table. S3**. Table of imputed genes

| **Official gene symbol** | **Ref (hg19) seq** | **Description** |
| --- | --- | --- |
| **TMEM98** | NM_015544 | Homo sapiens transmembrane protein 98 (TMEM98), transcript variant 1, mRNA. |
| **MYO1D** | NM_015194 | Homo sapiens myosin ID (MYO1D), mRNA. |
| **TMEM98** | NM_001033504 | Homo sapiens transmembrane protein 98 (TMEM98), transcript variant 2, mRNA. |
| **SPACA3** | NM_173847 | Homo sapiens sperm acrosome associated 3 (SPACA3), mRNA. |
| **ASIC2** | NM_183377 | Homo sapiens acid-sensing (proton-gated) ion channel 2 (ASIC2), transcript variant MDEG2, mRNA. |
| **ASIC2** | NM_001094 | Homo sapiens acid-sensing (proton-gated) ion channel 2 (ASIC2), transcript variant MDEG1, mRNA. |
| **AA06** |  | Homo sapiens uncharacterized LOC100506677 (AA06), non-coding RNA. |
| **RP11-31I22.3** |  | Homo sapiens cDNA FLJ32755 fis, clone TESTI2001725. |

Genes imputed with official symbol on the reference genome Ch37 (hg19), name of transcript and description.

**Table. S4.** Summary of LD-based GWAS trait analysis

| **GWAS Trait** | **PMID** | **SNP id** | **Position (GRCh37)** | **r^2^** | **P-value** |
| --- | --- | --- | --- | --- | --- |
| Schizophrenia | 26198764 | rs62064224 | chr17:30635115 | 0.32 | <0.001 |
| Smoking initiation (ever regular vs never regular) | 30643251 | rs2344976 | chr17:30685935 | 0.66 | <0.001 |
| Smoking initiation (ever regular vs never regular) (MTAG) | 30643251 | rs9896320 | chr17:30689814 | 0.66 | <0.001 |
| Smoking initiation (MTAG) | 37156939 | rs2286645 | chr17:30696292 | 0.68 | <0.001 |
| Schizophrenia | 35396580 | rs62065460 | chr17:30715007 | 0.41 | <0.001 |
| Schizophrenia | 33169155 | rs62065467 | chr17:30760318 | 0.40 | <0.001 |
| Systolic blood pressure | 30595370 | rs4794923 | chr17:30763009 | 0.68 | <0.001 |
| Systolic blood pressure | 30224653 | rs9899540 | chr17:30777924 | 0.66 | <0.001 |
| Systolic blood pressure | 38689001 | rs9899540 | chr17:30777924 | 0.66 | <0.001 |
| Pulse pressure | 38689001 | rs9899540 | chr17:30777924 | 0.66 | <0.001 |
| Smoking initiation | 36477530 | rs2302276 | chr17:30791503 | 0.69 | <0.001 |
| Externalizing behaviour (multivariate analysis) | 34446935 | rs4132610 | chr17:30801441 | 0.69 | <0.001 |
| Reactive lymphocyte percentage of white cells | 37596262 | rs28685302 | chr17:30846334 | 0.26 | <0.001 |
| Reactive lymphocyte count | 37596262 | rs28685302 | chr17:30846334 | 0.26 | <0.001 |

LD-tissue-specific analysis of rs756787. Variables reported are GWAS trait involved found in literature, PMID as reference of publication (second column), SNP id according to Ch37 (hg19, r^2^ as a measure of association of each SNP with rs756787 and correlation p-value.

**Table S5.** Summary of LD-based tissue-specific analysis

| **Gene** | **Gencode ID** | **Tissue** | **Non-effect Allele Freq** | **Effect Allele Freq** | **Effect Size** | **P-value** |
| --- | --- | --- | --- | --- | --- | --- |
| lncRNA | ENSG00000274341.1 | Cells - Cultured fibroblasts | T=0.348 | C=0.652 | 0.15 | <0.001 |
| C17orf75 | ENSG00000108666.9 | Cells - Cultured fibroblasts | T=0.348 | C=0.652 | 0.14 | <0.001 |
| lncRNA | ENSG00000274341.1 | Lung | T=0.348 | C=0.652 | 0.20 | <0.001 |
| lncRNA | ENSG00000266385.1 | Lung | T=0.348 | C=0.652 | 0.24 | <0.001 |
| RHBDL3 | ENSG00000141314.12 | Brain - Putamen (basal ganglia) | T=0.348 | C=0.652 | -0.46 | <0.001 |
| PSMD11 | ENSG00000279762.3 | Testis | T=0.348 | C=0.652 | 0.29 | <0.001 |
| C17orf75 | ENSG00000108666.9 | Testis | T=0.348 | C=0.652 | 0.17 | <0.001 |
| CDK5R1 | ENSG00000176749.8 | Testis | T=0.348 | C=0.652 | -0.19 | <0.001 |
| PSMD11 | ENSG00000108671.9 | Testis | T=0.348 | C=0.652 | 0.48 | <0.001 |
| CDK5R1 | ENSG00000176749.8 | Whole Blood | T=0.348 | C=0.652 | 0.15 | <0.001 |
| lncRNA | ENSG00000274341.1 | Brain - Cerebellum | T=0.348 | C=0.652 | 0.31 | <0.001 |
| RHBDL3 | ENSG00000141314.12 | Brain - Hippocampus | T=0.348 | C=0.652 | -0.29 | <0.001 |
| lncRNA | ENSG00000266718.1 | Pituitary | T=0.348 | C=0.652 | 0.33 | <0.001 |
| lncRNA | ENSG00000266718.1 | Muscle - Skeletal | T=0.348 | C=0.652 | 0.27 | <0.001 |
| RHBDL3 | ENSG00000141314.12 | Brain - Hypothalamus | T=0.348 | C=0.652 | -0.36 | <0.001 |
| RHBDL3 | ENSG00000141314.12 | Brain - Cortex | T=0.348 | C=0.652 | -0.26 | <0.001 |
| MYO1D | ENSG00000176658.16 | Artery - Aorta | T=0.348 | C=0.652 | -0.13 | <0.001 |
| lncRNA | ENSG00000274341.1 | Skin - Not Sun Exposed (Suprapubic) | T=0.348 | C=0.652 | 0.17 | <0.001 |
| RHBDL3 | ENSG00000141314.12 | Brain - Caudate (basal ganglia) | T=0.348 | C=0.652 | -0.33 | <0.001 |
| RHBDL3 | ENSG00000141314.12 | Brain - Nucleus accumbens (basal ganglia) | T=0.348 | C=0.652 | -0.49 | <0.001 |
| PSMD11 | ENSG00000108671.9 | Nerve - Tibial | T=0.348 | C=0.652 | 0.11 | <0.001 |
| PSMD11 | ENSG00000108671.9 | Artery - Tibial | T=0.348 | C=0.652 | 0.11 | <0.001 |
| MYO1D | ENSG00000176658.16 | Artery - Tibial | T=0.348 | C=0.652 | -0.10 | <0.001 |
| lnRNA | ENSG00000274341.1 | Skin - Sun Exposed (Lower leg) | T=0.348 | C=0.652 | 0.15 | <0.001 |
| lnRNA | ENSG00000266718.1 | Skin - Sun Exposed (Lower leg) | T=0.348 | C=0.652 | 0.19 | <0.001 |
| lnRNA | ENSG00000274341.1 | Stomach | T=0.348 | C=0.652 | 0.30 | <0.001 |

LD-tissue specific analysis for query variant rs756787. Ensemble code is reported for association with tissue-specific genes higher than 0.8 (r^2^>0.8), along with non-effect allele and effect allele frequencies, effect size and p-value.
